# The Association of Renal Function and Plasma Metals Modified by EGFR and TNF-α Gene Polymorphisms in Metal Industrial Workers and General Population

**DOI:** 10.3390/ijerph18178965

**Published:** 2021-08-25

**Authors:** Tzu-Hua Chen, Joh-Jong Huang, Hsiang-Ying Lee, Wei-Shyang Kung, Kuei-Hau Luo, Jia-Yi Lu, Hung-Yi Chuang

**Affiliations:** 1 Department of Public Health, College of Health Sciences, Kaohsiung Medical University, Kaohsiung 80708, Taiwan; 980264@kmuh.org.tw (T.-H.C.); u105570008@kmu.edu.tw (J.-Y.L.); 2 Department of Family Medicine, Kaohsiung Municipal Ta-Tung Hospital, Kaohsiung 80145, Taiwan; 3 Department of Family Medicine, Kaohsiung Medical University Hospital, Kaohsiung 80708, Taiwan; jjhua511227@gmail.com; 4 Department of Urology, Kaohsiung Medical University Hospital, Kaohsiung 80708, Taiwan; 1008011@ms.kmuh.org.tw; 5 Department of Urology, School of Medicine, College of Medicine, Kaohsiung Medical University, Kaohsiung 80708, Taiwan; 6 Department of Urology, Kaohsiung Municipal Ta-Tung Hospital, Kaohsiung 80145, Taiwan; 7 Graduate Institute of Clinical Medicine, College of Medicine, Kaohsiung Medical University, Kaohsiung 80708, Taiwan; 8 Department of Pediatrics, Chien Shin Hospital, Kaohsiung 80143, Taiwan; u104862002@kmu.edu.tw; 9 Graduate Institute of Medicine, College of Medicine, Kaohsiung Medical University, Kaohsiung 80708, Taiwan; u107800007@kmu.edu.tw; 10 Department of Environmental and Occupational Medicine, Kaohsiung Medical University Hospital, Kaohsiung 80708, Taiwan; 11 Ph.D. Program in Environmental and Occupational Medicine, Research Center for Environmental Medicine, College of Medicine, Kaohsiung Medical University, Kaohsiung 80708, Taiwan

**Keywords:** health risk assessment, toxic metals, EGFR, TNF-α, single nucleotide polymorphism (SNP), renal function, environmental strategy, Taiwan Biobank (TWB)

## Abstract

Exposure to metals may be associated with renal function impairment, but the effect modified by genetic polymorphisms was not considered in most studies. Epidermal growth factor receptor (EGFR) and tumor necrotic factor-α (TNF-α) play important roles in renal hemodynamics, and they have been reported to be associated with some renal diseases. The aim of our research is to explore whether genetic variations in EGFR and TNF-α have influence on renal function under exposure to various metals. This cross-sectional study consisted of 376 metal industrial workers, 396 participants of Taiwan Biobank, and 231 volunteers of health examinations. We identified 23 single nucleotide polymorphisms (SNPs) on the EGFR gene and 6 SNPs on the TNF-α gene, and we also measured their plasma concentration of cobalt, copper, zinc, selenium, arsenic, and lead. Multiple regression analysis was applied to investigate the association between various SNPs, metals, and renal function. Our results revealed some protective and susceptible genotypes under occupational or environmental exposure to metals. The individuals carrying EGFR rs2280653 GG might have declined renal function under excessive exposure to selenium, and those with EGFR rs3823585 CC, rs12671550 CC, and rs4947986 GG genotypes might be susceptible to lead nephrotoxicity. We suggest the high-risk population to prevent renal diseases.

## 1. Introduction

Renal function impairment may progress to chronic kidney disease (CKD), one of the public health issues worldwide with a global prevalence of 9.1% [1]. The leading causes of CKD are diabetes and hypertension, whereas exposure to heavy metals and genetic factors also contribute to CKD [2]. Early identification and preventive strategies deserve attention in order to reduce the burden of this non-communicable disease. 

Metals are widely used in industries, while the general public may also be exposed to multiple metals in the environment. The impact of metals on the kidney was historically characterized in occupational and general populations, and some metals were reported to have nephrotoxicity [3,4,5,6]. Nevertheless, the influence of genetic factors was not considered in these studies. Owing to increasing knowledge about gene–environment interactions [7], it is of urgency to discover the susceptible genotypes to avoid the harmful effects of metals.

The epidermal growth factor receptor (EGFR) is a member of the transmembrane receptors tyrosine kinase family, mediating cellular responses in many tissues and organs [8]. EGFR is expressed in the kidney, and EGFR activation plays a vital role in renal hemodynamics and electrolyte balance [9,10]. Korean research proposed that EGFR gene polymorphisms may be associated with end stage renal disease (ESRD) and acute renal allograft rejection [11]. However, it is somewhat contradictory because both beneficial and detrimental effects may occur [9,10]. 

Tumor necrosis factor-α (TNF-α), a proinflammatory cytokine, is mainly produced by T-lymphocytes; moreover, some other cells, such as renal tubular epithelial and mesangial cells may also secrete TNF-α [12]. The renal actions of TNF-α include the regulation of hemodynamic and excretory function in the kidney, by inducing renal vasoconstriction and hypofiltration [12,13]. Some studies have demonstrated that TNF-α gene polymorphisms are associated with acute kidney injury (AKI), chronic renal failure (CRF), and ESRD [14,15]. Nevertheless, the relationship between metal-related renal function change and TNF-α gene polymorphisms has not been published.

Cobalt (Co), copper (Cu), zinc (Zn), and selenium (Se) are essential trace elements in human beings, but they may cause an injury in high concentrations [16,17,18,19]. On the other hand, arsenic (As) and lead (Pb) are toxic metalloids/metals. All of them are universal metals in both working and living environments. The aim of our research is to explore whether the associations between metals and kidney function could be modified by EGFR and TNF-α genetic polymorphisms.

## 2. Materials and Methods

### 2.1. Study Population and Renal Function Measurement

The subjects of this cross-sectional study consisted of 376 metal industrial workers and 627 non-metal industrial workers. We obtained the data of 376 metal workers with an annual health examination in Kaohsiung Medical University Hospital, a medical center in southern Taiwan. Among the non-metal workers, the data of 396 subjects were gathered from Taiwan Biobank (TWB), and 231 subjects were recruited from the volunteers receiving health examinations in the hospital. TWB is the largest biobank supported by the government in Taiwan, with the purpose of establishing lifestyle and genomic databases of Taiwanese residents [20]. All participants received physical examinations and face-to-face interviews, and they also provided blood samples. The questions of the interviews included occupation, medical history, current drug use, and the habit of smoking. All procedures were granted by the Kaohsiung Medical University Hospital Institutional Review Board (approval number: KMUHIRB-E(I)-20150259), and all individuals signed the approved informed consent form. 

The age of individuals in our study ranged from 18 to 65. We excluded the subjects with cancer, nephritis, and CKD to avoid the impact of these diseases. We calculated estimated glomerular filtration rate (eGFR) by the Chronic Kidney Disease Epidemiology Collaboration (CKD-EPI) equation and Modification of Diet in Renal Disease (MDRD) equation, and there was no difference in our results. Therefore, we used the CKD-EPI equation for our eGFR definition because a systematic review supported its strength at higher GFR ranges [21].

### 2.2. Analyses of Plasma Metals

Plasma concentrations of all metals and elements were analyzed by inductively coupled plasma mass spectrometry (ICPMS, Thermo Scientific XSERIES 2) at the laboratory in Kaohsiung Medical University. Radio Corporation of America (RCA) cleaning was used on all equipment in the laboratory. For sample preparation, 1% HNO3 was added into plasma samples to make a 1:10 dilution and then we let it sit for 10 min. For checking of high linearity, ICP-MS calibration standard solution (AccuStandard, MES-04-1) was diluted to 0.1, 0.2, 0.5, 1, 2, 5, 10, 20, 50, 100, 200, 500, 1000, 2000, and 3000 μg/L to estimate the calibration curve, and each element was consistent with the curve with a high correlation coefficient (r > 0.995). We checked plasma concentrations of Co, Cu, Zn, Se, As, and Pb. Before analyzing the unknown concentrations, we conducted quality assurance (QA) and quality control (QC) to ensure precision and accuracy. QA was to analyze standard reference materials (SRMs). To ensure the consistence of laboratory tests, we took random SRMs to conduct repeated analysis, and each result had to fit the curve between 90% and 110%. QC was to ensure the stability of the system by triple testing SRMs, of which their coefficient of variance (CV) should be less than 3%.

### 2.3. Genotyping

Single nucleotide polymorphism (SNP) genotyping was performed using the custom TWB chips and run on the Axiom Genome-Wide Array Plate System (Affymetrix, Santa Clara, CA, USA). TWB1 array was designed for Taiwan’s Han Chinese and released in April 2013. Furthermore, TWB2 array released in August 2018 was based on the experience of TWB1 use and designed for further clinical use. There were approximately 653k autosomal SNPs and 752k SNPs which could be genotyped in TWB1 and TWB2 arrays, respectively. There were about 105k overlapping SNPs in two TWB arrays [22].

The individuals of the non-metal worker group were genotyped using TWB1 chips, which revealed 73 SNPs on the EGFR gene and 6 SNPs on the TNF-α gene. On the other hand, the individuals of the metal worker group were genotyped using TWB2 chips, which revealed 229 SNPs on the EGFR gene and 31 SNPs on the TNF-α gene. Regarding QC in the genetic study, we used PLINK 1.9 to calculate the genome-wide identity by descent (IBD) to ensure the unrelatedness of all DNA samples, and we excluded the individuals with IBD > 0.1875 [23,24]. We also excluded the SNPs with a genotyping rate < 95% and Hardy–Weinberg test *p*-values < 10^−6^ [24,25]. Moreover, we used (N-O)/N to calculate mean heterozygosity, where N was the number of non-missing genotypes and O was the observed number of homozygous genotypes for a given subject. Individuals with more than 3 standard deviations from mean heterozygosity were excluded due to the possibility of DNA sample contamination or inbreeding [24]. After the QC process, we compared the SNPs of the EGFR and TNF-α gene which were genotyped in TWB1 and TWB2 arrays, respectively. Between these two arrays, 23 SNPs on the EGFR gene and 6 SNPs on the TNF-α gene were overlapped, and thus a total of 29 SNPs were kept in our analysis.

### 2.4. Statistical Analyses

Making a comparison between non-metal workers and metal workers, we used the χ2 test for categorical variables and Student’s t test for continuous variables. To investigate the association between gene polymorphisms, plasma metal concentrations, and renal function, we applied multiple regression analysis. Covariates in all models included age, gender, body mass index (BMI), consumption of cigarettes, diabetes, and hypertension history. The dependent variable was eGFR calculated by the CKD-EPI equation.

Initially, to test whether gene polymorphisms were significantly associated with renal function, we regressed eGFR on each of the 29 SNPs with adjustment for covariates:

eGFR = β_0_ + β_SNP*,i*_ SNP*_i_* + β_c_ Covariates + ε, *i* = 1, ..., 29 (1)

where SNP*_i_* is the number of minor alleles at the *i*th SNP (0, 1, or 2) and ε is the error term. By testing H_0_: β_SNP*,i*_ = 0 versus H_1_: β_SNP*,i*_ ≠ 0, we obtained beta coefficients and 95% confidence intervals (CIs) for associations between the *i*th SNP and eGFR. 

We also examined the relationship between 6 plasma metal concentrations and eGFR:

eGFR = β_0_ + β_M,*j*_ M*_j_* + β_c_ Covariates + ε, *j* = 1, ..., 6, (2)

where M is the plasma concentration of Co, Cu, Zn, Se, As, and Pb, respectively.

Then, we regressed eGFR on every metal and every SNP, and in total, 174 regression models were built as follows:

eGFR = β_0_ + β_M,*j*_ M*_j_* + β_SNP,1*i*_ SNP_1*i*_ + β_SNP,2*i*_ SNP_2*i*_ + β_c_ Covariates + ε, (3)

where two dummy variables were used in the genotypes. We set major allele homozygous genotypes as reference. We set SNP_1*i*_ = 1 for heterozygous genotypes and SNP_1*i*_ = 0, otherwise. Similarly, we set SNP_2*i*_ = 1 for minor allele homozygous genotypes and SNP_2*i*_ = 0, otherwise.

Finally, we considered the interaction between metals and SNPs, and we built 174 regression models:

eGFR = β_0_ + β_M,*j*_ M*_j_* + β_SNP,1*i*_ SNP_1*i*_ + β_SNP,2*i*_ SNP_2*i*_ + β_int,1*i*_ M*_j_* × SNP_1*i*_ +β_int,2*i*_ M*_j_* × SNP_2*i*_ + β_c_ Covariates + ε. (4)

The analyses were executed by the SAS package (version 9.4; SAS Institute, Cary, NC, USA), and a two-tailed *p*-value < 0.05 indicated statistical significance.

## 3. Results

### 3.1. Basic Information and Analysis of Study Population

Table 1 shows the comparison of demographic characteristics, physical and biochemical parameters, and plasma metal concentrations between the non-metal industrial workers and metal industrial workers. No significant difference was found in gender, age, uric acid, and eGFR between the two groups. Those in the metal workers group had higher smoking prevalence, BMI, systolic blood pressure (SBP), sugar, total cholesterol, and alanine aminotransferase (ALT). In addition, they also had higher plasma metal concentrations.

The average duration of employment in our metal industrial workers was 14.16 ± 11.51 years. The duration was associated with renal function (β = −0.54, *p* < 0.01). Nevertheless, renal function declined with increasing age, and there was collinearity between age and duration (Pearson correlation coefficient = 0.75, *p* < 0.001). Therefore, after adjustment for age, the duration had no association with renal function (β = −0.05, *p* = 0.51).

The mean duration of metal workers was 14.09 ± 12.68 years in males and 14.23 ± 10.36 years in females, and there was no gender difference in metal exposure (*p* = 0.91). The average eGFR was 104.02 ± 11.68 mL/min/1.73m^2^ in males and 105.34 ± 13.58 mL/min/1.73 m^2^ in females, and there was no gender difference in renal function (*p* = 0.09). With regard to SNPs, there was gender difference in EGFR rs2302535 A > C, rs11238349 G > A, rs2472520 G > C, and rs1800610 G > A, and the multiple regression analysis on each SNP was performed respectively with adjustment for gender.

### 3.2. The Association between Renal Function and SNPs

**Figure 1:**
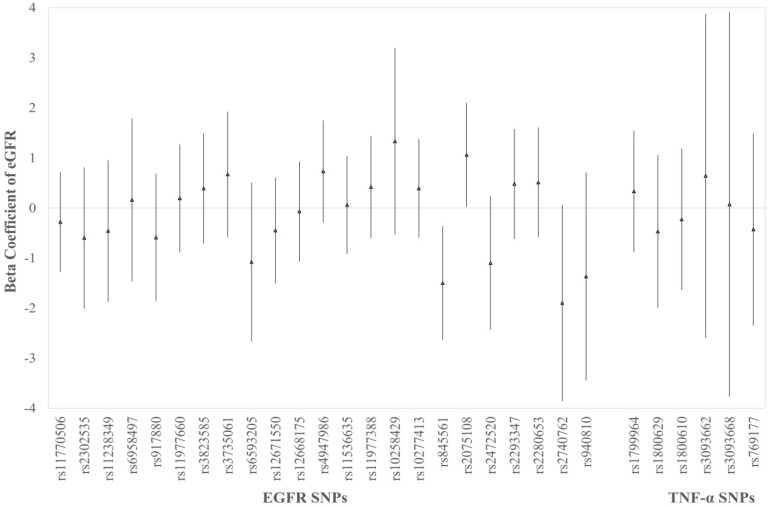
Beta coefficients and 95% confidence intervals (CIs) for associations between eGFR and 29 SNPs, respectively. Adjusted for age, gender, BMI, smoking, diabetes, and hypertension. (rs1799964, rs1800629, rs1800610, rs3093662, rs3093668, and rs769177 are TNF-α SNPs, and the others are EGFR SNPs.)

According to Equation (1), regression of eGFR on each SNP showed a decline in rs845561 C > T (β = −1.5, 95% CI: −2.64, −0.36) and an elevation in rs2075108 A > G (β = 1.06, 95% CI: 0.02, 2.1) (Figure 1). 

### 3.3. The Association between Renal Function and Metals

**Figure 2:**
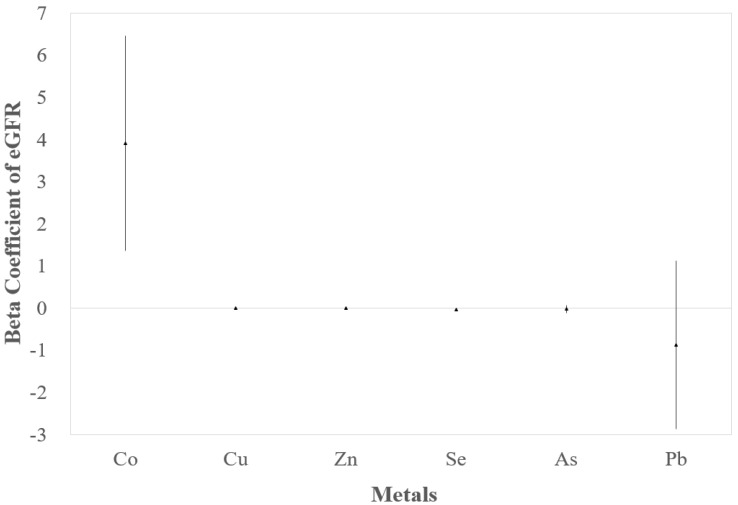
Beta coefficients and 95% CIs for associations between eGFR and 6 metals, respectively. Adjusted for age, gender, BMI, smoking, diabetes, and hypertension. Beta coefficient of eGFR of Se was −0.03 (95% CI: −0.04, −0.02). Cu, Zn, and as had no association with eGFR, and the beta coefficients were small (β = 0.002, −0.001, −0.015, respectively).

According to Equation (2), regression of eGFR on each metal showed an increase in Co (β = 3.92, 95% CI: 1.37, 6.47) and a decrease in Se (β = −0.03, 95% CI: −0.04, −0.02) (Figure 2).

### 3.4. The Associations between Renal Function, Metals, and SNPs

**Figure 3:**
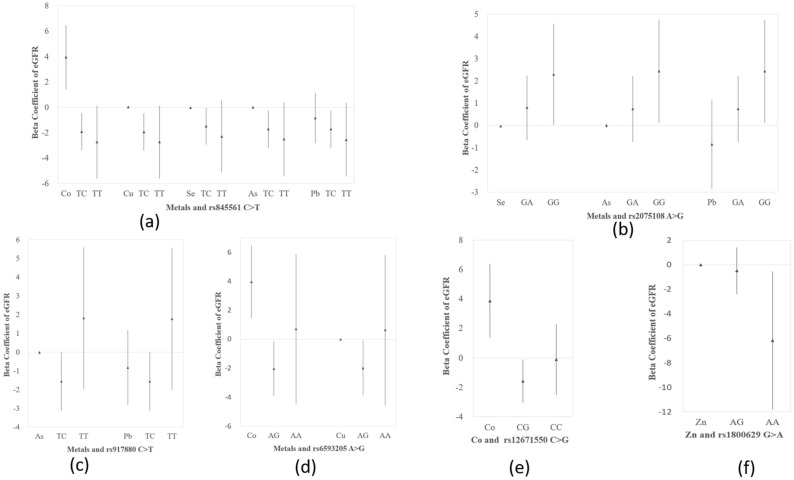
Beta coefficients and 95% CIs for associations between eGFR and metals and SNPs, respectively. The regression model was built as eGFR = β_0_ + β_M,*j*_ M*_j_* + β_SNP,1*i*_ SNP_1*i*_ + β_SNP,2*i*_ SNP_2*i*_ + β_c_ Covariates + ε. Adjusted covariates included age, gender, BMI, smoking, diabetes, and hypertension. (**a**) Metals and rs845561 C > T. Regression of eGFR on metals was increased in Co (β = 3.95, 95% CI: 1.42, 6.48) and decreased in Se (β = −0.03, 95% CI: −0.04, −0.02). Genotype TC was associated with reduced eGFR, β = −1.93 (95% CI: −3.4, −0.46) adjusting for Co, β = −1.94 (−3.41, −0.47) adjusting for Cu, β = −1.5 (−2.95, −0.05) adjusting for Se, β = −1.73 (−3.2, −0.26) adjusting for As, and β = −1.72 (−3.19, −0.25) adjusting for Pb. (**b**) Metals and rs2075108 A > G. Regression of eGFR on metals was decreased in Se (β = −0.03, 95% CI: −0.04, −0.02). Genotype GG was associated with increased eGFR, β = 2.29 (0.02, 4.56) adjusting for Se, β = 2.43 (0.12, 4.74) adjusting for As, and β = 2.43 (0.12, 4.74) adjusting for Pb. (**c**) Metals and rs917880 C > T. Genotype TC was associated with decreased eGFR, β = −1.57 (−3.14, −0.002) adjusting for As, and β = −1.57 (−3.14, −0.002) adjusting for Pb. (**d**) Metals and rs6593205 G > A. Regression of eGFR on metals was elevated in Co (β = 3.98, 95% CI: 1.45, 6.51). Genotype AG was associated with declined eGFR, β = −2.02 (−3.9, −0.14) adjusting for Co, and β = −1.98 (−3.86, −0.1) adjusting for Cu. (**e**) Metal and rs12671550 C > G. Regression of eGFR on metals was elevated in Co (β = 3.87, 95% CI: 1.34, 6.4). Genotype CG was associated with reduced eGFR, β = −1.57 (−3.04, −0.1) adjusting for Co. (**f**) Metal and rs1800629 G > A. Genotype AA was associated with declined eGFR, β = −6.19 (−11.82, −0.56) adjusting for Zn.

According to Equation (3), the associations between eGFR and metals and SNPs were plotted as Figure 3. In rs845561 C > T, regression of eGFR on metals showed an increase in Co (β = 3.95, 95% CI: 1.42, 6.48) and a decrease in Se (β = −0.03, 95% CI: −0.04, −0.02). Compared with the reference genotype CC, genotype TC was associated with reduced eGFR. The beta coefficients of genotype TC were −1.93 (95% CI: −3.4, −0.46) adjusting for Co, −1.94 (95% CI: −3.41, −0.47) adjusting for Cu, −1.5 (95% CI: −2.95, −0.05) adjusting for Se, −1.73 (95% CI: −3.2, −0.26) adjusting for As, and −1.72 (95% CI: −3.19, −0.25) adjusting for Pb (Figure 3a). In rs2075108 A > G, regression of eGFR on metals showed a decrease in Se (β = −0.03, 95% CI: −0.04, −0.02). Compared with genotype AA, genotype GG was associated with increased eGFR. The beta coefficients of genotype GG were 2.29 (95% CI: 0.02, 4.56) adjusting for Se, 2.43 (95% CI: 0.12, 4.74) adjusting for As, and 2.43 (95% CI: 0.12, 4.74) adjusting for Pb (Figure 3b). In rs917880 C > T, compared with genotype CC, genotype TC was associated with decreased eGFR. The beta coefficients of genotype TC were −1.57 (95% CI: −3.14, −0.002) adjusting for As, and −1.57 (95% CI: −3.14, −0.002) adjusting for Pb (Figure 3c). In rs6593205 A > G, regression of eGFR on metals showed an elevation in Co (β = 3.98, 95% CI: 1.45, 6.51). Compared with genotype GG, genotype AG was associated with declined eGFR. The beta coefficients of genotype AG were −2.02 (95% CI: −3.9, −0.14) adjusting for Co, and −1.98 (95% CI: −3.86, −0.1) adjusting for Cu (Figure 3d). In rs12671550 C > G, regression of eGFR on metals showed an elevation in Co (β = 3.87, 95% CI: 1.34, 6.4). Compared with genotype GG, genotype CG was associated with reduced eGFR adjusting for Co, and the beta coefficient was −1.57 (95% CI: −3.04, −0.1) (Figure 3e). In rs1800629 G > A, compared with genotype GG, genotype AA was associated with declined eGFR adjusting for Zn, and the beta coefficient was −6.19 (95% CI: −11.82, −0.56) (Figure 3f).

### 3.5. The Interactions between Metals and SNPs on Renal Function

**Figure 4:**
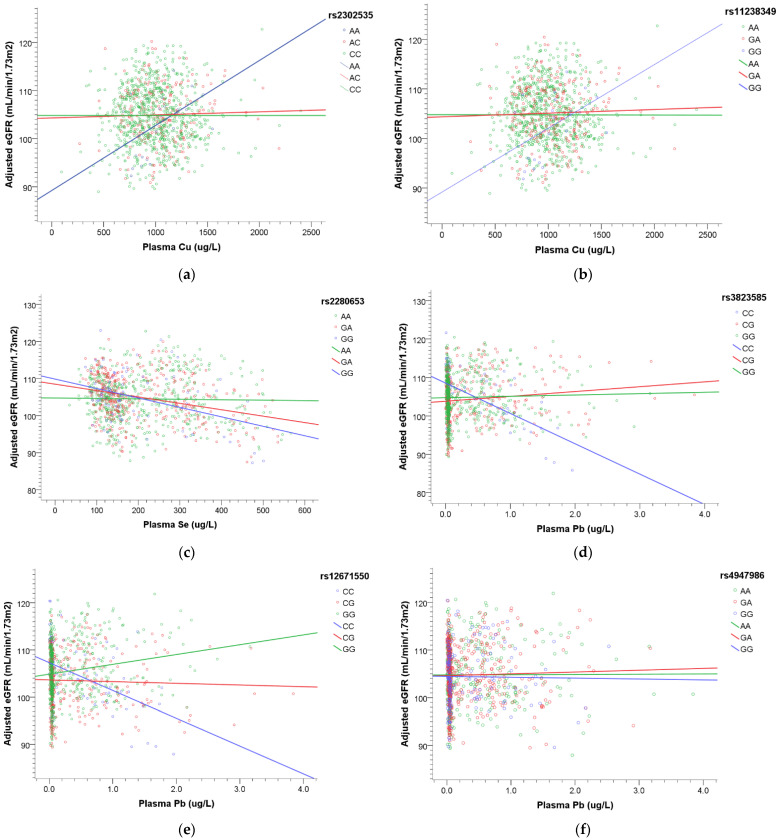
The association of plasma metals with renal function modified by SNPs. The regression model was built as eGFR = β_0_ + β_M,*j*_ M*_j_* + β_SNP,1*i*_ SNP_1*i*_ + β_SNP,2*i*_ SNP_2*i*_ + β_int,1*i*_ M*_j_* × SNP_1*i*_ +β_int,2*i*_ M*_j_* × SNP_2*i*_ + β_c_ Covariates + ε. Adjusted covariates included age, gender, BMI, smoking, diabetes, and hypertension. (**a**) Increasing plasma Cu with elevated eGFR in rs2302535 AA genotype. (**b**) Increasing plasma Cu with elevated eGFR in rs11238349 GG genotype. (**c**) Increasing plasma Se with declined eGFR in rs2280653 GG and GA genotype, especially a steeper slope noted in GG genotype. (**d**) A declined eGFR was noted in rs3823585 CC genotype with increasing plasma Pb. (**e**) A declined eGFR was noted in rs12671550 CC genotype with increasing plasma Pb. (**f**) Increasing plasma Pb with mild decreased eGFR in rs4947986 GG genotype.

According to Equation (4), we plotted Figure 4 to express the interactions between metals and SNPs that influence renal function. With an increase in plasma Cu concentration, elevated eGFR was noted in rs2302535 AA and rs11238349 GG genotype (Figure 4a,b). When the plasma Se level increased, renal function declined in rs2280653 GG and GA genotype (Figure 4c). When the plasma Pb concentration increased, declined eGFR was noted in rs3823585 CC, rs12671550 CC, and rs4947986 GG genotype (Figure 4d–f).

## 4. Discussion

Nephrotoxicity of some metals was reported in prior literature, but the influence of gene polymorphisms was rarely considered [3,4,5]. In our study, we investigated the associations between EGFR and TNF-α gene polymorphisms, plasma metal concentrations, and renal function.

Exposure to multiple metals may lead to some changes in renal function. Our research revealed that cobalt might be protective towards kidney function. Cobalt toxicity drew much attention owing to a wide use in dietary supplements and Co-containing alloys in medical devices. Although there were doubts regarding adverse effects on the kidney, no deterioration of renal function was observed in a 10-year follow-up study in the patients after metal-on-metal hip arthroplasty [26]. In addition, cobalt administration was reported to induce renoprotective gene expression and improve ischemic renal injury in rats [27]. Further in vitro study revealed that cobalt chloride might protect against renal inflammation by reducing oxidative stress in human proximal tubular epithelial cells [28].

EGFR exists in a range of tissues, and abnormal EGFR expression may trigger downstream signaling leading to cancers and many other diseases [8]. EGFR activation by metals has been supposed to be involved in some neurodegenerative diseases [29,30]. To date, genetic studies have demonstrated that EGFR polymorphisms may modify the risk for head and neck squamous cell carcinoma (HNSCC), lung cancer, renal cancer, and so on [31,32,33]. Nevertheless, there were no studies about the relationship between metals, kidney function, and EGFR polymorphisms. Our study showed that EGFR rs845561 and rs2075108 were associated with renal function. More specifically, we proposed that rs845561 TC might be a susceptible genotype and rs2075108 GG might be a protective genotype with adjustment for plasma metal levels. Similarly, Fung et al. reported that rs845561 TC and CC genotypes were associated with increasing HNSCC risk [31], suggesting that rs845561 may modify the risk for human diseases, but the mechanism was not clear. In addition, our results revealed that EGFR rs917880 TC, rs6593205 AG, and rs12671550 CG might be susceptible genotypes for decreased renal function with adjustment for plasma metal concentration. These genetic variants have not been found to have any significant association with human health.

TNF-α gene polymorphisms are associated with autoimmune diseases, infection, cancers, and many other diseases, and TNF-α rs1800629 has been the most studied polymorphism [34,35]. Our results revealed that rs1800629 AA genotype had a significant association with renal function decrease with adjustment for plasma zinc level. This finding was in line with most previous studies. A systematic review and meta-analysis showed that rs1800629 GA and AA genotypes had a higher trend of AKI in Asians [14]. The homozygous AA genotype was found to have a relationship with CRF and ESRD [15], and A allele had a higher risk of acute rejection in first kidney transplant subjects [36]. 

We observed that some metals interact with EGFR and TNF-α SNPs, influencing the change of renal function. The normal values of plasma copper concentration are about 1000 μg/L, ranging up to about 1500 μg/L [37]. Some cases of copper poisoning suffered from anuria or oliguria, and even renal failure [37]. Our result showed that the EGFR rs2302535 AA genotype and rs11238349 GG genotype played protective roles under higher plasma copper concentration. According to prior studies, EGFR rs2302535 had an association with radiation-induced esophagitis in lung cancer patients [38], and rs11238349 might be involved in the pathway in the development of prostate cancer [39], but the relationship between these two SNPs and the kidney has not been indicated. The reference range of plasma selenium concentration is 40 to 200 μg/L in healthy adults [40], and our results revealed that the individuals carrying EGFR rs2280653 GG and GA genotypes might be susceptible to renal dysfunction beyond normal physiological selenium levels. Lead-related damage to kidneys was one of the most widely studied metals [41,42]. To our knowledge, polymorphisms of the δ-aminolevulinic acid dehydratase (ALAD) gene and vitamin D receptor (VDR) gene were reported to modify the association between lead and renal function [43,44], but no other genes were studied subsequently. The selected genes were different in our research, and we proposed that higher lead exposure might be associated with worse renal function in EGFR rs3823585 CC, rs12671550 CC, and rs4947986 GG genotype. In our findings, we observed that the susceptible genotypes were minor allele homozygous genotypes in our study population. We supposed it is reasonable because the ratio of susceptible individuals might decrease in the evolution.

Oxidative stress was supposed to be related to renal function impairment. The interactions between metals and genotypes are very complicated, and the mechanism of protective or harmful effect remains unclear. Moreover, people may be exposed to multiple metals simultaneously in the environment, resulting in synergistic or antagonistic interactions. Further studies are necessary to investigate the kinetic interactions in human beings.

This is pioneer research to explore the effect of EGFR and TNF-α gene polymorphisms on the association between metals and renal function. Moreover, our study subjects included metal industrial workers and participants of TWB and health examinations. This is also the first study to observe the impact of metals on renal function in both the occupational and general population. There are several limitations. First, this is a cross-sectional study, so we could not follow up the long-term change of renal function. Secondly, there were no data about urine protein. However, the individuals with CKD history were excluded from this research, so our study subjects were free of persistent proteinuria. Although the exclusion criteria of CKD might mistakenly delete the individuals with CKD induced by metal toxicity, we still obtained significant results, and thus our findings could be believable. Third, the prevalence of diabetes was higher in our metal workers (6.9% in metal group versus 1.8% in non-metal group, *p* < 0.001), but it was still in line with the average prevalence of diabetes in Taiwan. The ratio of hypertension had no significant difference in the two groups (15.2% in metal group versus 11.2% in non-metal group, *p* = 0.08). We have included diabetes and hypertension history as covariates in multiple regression analysis. On the other hand, the average eGFR was 104.67 ± 20.16 mL/min/1.73m^2^ in our research, probably owing to the healthy worker effect [45], and the participants of the health examinations put emphasis on their health. Nevertheless, we still discovered some significant changes of renal function under the complex interaction between genetic polymorphisms and metals.

## 5. Conclusions

We propose that EGFR and TNF-α gene polymorphisms may modify the effects of metals exerting on renal function. Our results revealed some protective and susceptible genotypes under occupational or environmental exposure to excessive metals. The individuals carrying EGFR rs2280653 GG genotype might have renal function impairment under excessive exposure to selenium, and those with EGFR rs3823585 CC, rs12671550 CC, and rs4947986 GG genotypes might be susceptible to lead nephrotoxicity. It is valuable in health risk assessments, and we need to educate the high-risk population to prevent kidney damage. Further prospective and large-scale research is warranted to confirm the association.

## Figures and Tables

**Table 1:** Comparison of demographic characteristics, physical and biochemical parameters, and plasma metal concentrations between the non-metal industrial workers and metal industrial workers.

Variable	Total *n* = 1003	Non-Metal Workers *n* = 627	Metal Workers *n* = 376	*p* Value
Gender				0.056
Male	514 (51.2)	334 (53.3)	180 (47.9)	
Female	489 (48.8)	293 (46.7)	196 (52.1)	
Smoking				<0.001
Yes	213 (21.2)	90 (14.4)	123 (32.7)	
No	790 (78.8)	537 (85.6)	253 (67.3)	
Age (year)	43.54 ± 10.03	43.16 ± 9.50	44.18 ± 10.83	0.117
BMI (kg/m^2^)	24.40 ± 3.96	24.01 ± 3.45	25.05 ± 4.62	<0.001
SBP (mmHg)	117.82 ± 16.62	115.00 ± 16.22	122.53 ± 16.23	<0.001
DBP (mmHg)	72.01 ± 11.32	72.74 ± 10.96	70.79 ± 11.81	0.008
Sugar (mg/dL)	94.69 ± 25.69	92.89 ± 18.38	97.70 ± 34.41	0.004
TC (mg/dL)	202.08 ± 37.41	197.81 ± 35.42	209.20 ± 39.54	<0.001
Uric acid (mg/dL)	5.75 ± 1.54	5.74 ± 1.55	5.76 ± 1.52	0.832
ALT (IU/L)	26.21 ± 19.77	24.97 ± 19.22	28.26 ± 20.53	0.011
Creatinine (mg/dL)	0.77 ± 0.17	0.78 ± 0.17	0.75 ± 0.16	0.009
eGFR^1^ (mL/min/1.73 m^2^)	104.67 ± 12.65	104.15 ± 12.26	105.53 ± 13.24	0.095
eGFR^2^ (mL/min/1.73 m^2^)	100.41 ± 20.16	99.56 ± 20.31	101.82 ± 19.85	0.086
Co (μg/L)	0.85 ± 0.29	0.78 ± 0.29	0.97 ± 0.26	<0.001
Cu (μg/L)	1003.54 ± 270.94	951.81 ± 266.22	1088.93 ± 257.00	<0.001
Zn (μg/L)	851.35 ± 276.60	792.91 ± 260.44	947.82 ± 275.82	<0.001
Se (μg/L)	207.41 ± 107.83	144.73 ± 45.47	311.91 ± 100.48	<0.001
As (μg/L)	6.07 ± 8.10	4.72 ± 3.32	8.31 ± 12.20	<0.001
Pb (μg/L)	0.34 ± 0.52	0.05 ± 0.04	0.83 ± 0.58	<0.001

Data are presented as *n*(%) or mean ± standard deviation. BMI—body mass index; SBP—systolic blood pressure; DBP—diastolic blood pressure; TC—total cholesterol; ALT—alanine aminotransferase; eGFR^1^—estimated glomerular filtration rate by the Chronic Kidney Disease Epidemiology Collaboration (CKD-EPI) equation; eGFR^2^—estimated glomerular filtration rate by the Modification of Diet in Renal Disease (MDRD) equation.

## Data Availability

The data from metal industrial workers and health examinations are available from the corresponding author. Restrictions apply to the availability of these data, which were used under license for this study. The data from Taiwan Biobank in this study can be applied from the Taiwan Biobank at https://www.twbiobank.org.tw/new_web_en/about-export.php (accessed on 20 August 2021).
